# The Effects of an Antimycotoxin Additive Based on Aluminosilicate and Marine Macroalgae in a Fumonisin-Contaminated Diet on the Performance and Health of Growing Lambs

**DOI:** 10.3390/ani16213342

**Published:** 2026-10-23

**Authors:** Davi Fernando Alba, Cristiano Tacca, Suelyn de Oliveira Marques, Tainara de Cezaro Alberti, Aline Gabriela Henrich, Eduardo Micotti Gloria, Aline Zampar, Aleksandro Schafer da Silva

**Affiliations:** 1 Programa de Pós-Graduação em Zootecnia, Universidade do Estado de Santa Catarina, Chapecó 89815-630, Brazil; davi.alba@hotmail.com (D.F.A.); suelyn.marques@edu.udesc.br (S.d.O.M.); tainara.cezaro@udesc.br (T.d.C.A.); aline.zampar@udesc.br (A.Z.); 2 Curso de Medicina Veterinária, Unidade Central de Educação Faem Faculdade (UCEFF), Chapecó 89812-214, Brazil; cistacca@gmail.com; 3 Departamento de Zootecnia, Universidade do Estado de Santa Catarina, Chapecó 89815-630, Brazil; 10470802901@edu.udesc.br; 4 Escola Superior de Agricultura Luiz de Queiroz (ESALQ), Universidade de São Paulo, São Paulo 05508-900, Brazil; emgloria@usp.br

**Keywords:** mycotoxins, antimycotoxin additive, animal health, oxidative stress, serum biochemistry, hematology, feed efficiency

## Abstract

Fumonisins are important feed contaminants that can impair animal health and productive performance. This study evaluated whether the inclusion of an antimycotoxin additive based on aluminosilicate and marine macroalgae could mitigate the adverse effects of dietary fumonisin in growing lambs. Thirty-two male Lacaune lambs were assigned to four groups (n = 8): control, control + additive, fumonisin, and fumonisin + additive. The additive was included at 3 kg/t of feed, and fumonisin was provided at 33 ppm. The experimental period lasted 42 days. Productive performance, serum biochemical parameters, oxidative status, and hematological variables were evaluated throughout the experiment. Fumonisin exposure impaired performance from days 15 to 42, reducing body weight gain, average daily gain, and feed efficiency and increasing feed conversion ratio, without affecting dry matter intake. Fumonisin also increased serum creatine kinase activity and altered cholesterol, gamma-glutamyl transferase, and aspartate aminotransferase, along with increasing thiobarbituric acid reactive substances and oxidant-related fluorescence. Time-dependent alterations were also observed in urea, ferritin, and hematological variables. Inclusion of the additive partially attenuated several fumonisin-associated responses, with Fumonisin + additive lambs showing performance and health-related variables closer to those of Control animals for several outcomes. Dietary fumonisin at 33.9 ppm adversely affected productive performance and was associated with biochemical, oxidative, and hematological alterations in growing lambs. Inclusion of the aluminosilicate- and marine macroalgae-based additive partially attenuated these responses, indicating potential for mitigating the biological consequences of fumonisin exposure, although complete protection was not achieved.

## Simple Summary

Dietary fumonisin exposure impaired growth performance and feed efficiency in growing lambs, without reducing dry matter intake. Fumonisin also altered serum biochemical parameters, increasing creatine kinase, AST, and GGT, and increased markers of oxidative imbalance, including TBARS and reactive oxygen species. Several hematological responses were also altered after prolonged exposure; for example, an increase in total leukocytes was observed in the group that received only fumonisin as well as a reduction in hematocrit. The aluminosilicate- and marine macroalgae-based additive attenuated several of these fumonisin-associated changes, with performance and health-related variables approaching those of control lambs. Thus, the additive showed potential to partially mitigate the biological effects of dietary fumonisin, although complete protection was not achieved.

## 1. Introduction

Mycotoxins are among the major challenges associated with animal feed safety and can adversely affect animal health and productive performance. These low-molecular-weight compounds are produced by fungi and can induce toxic responses when ingested by animals [1]. Mycotoxins may contaminate feedstuffs at different stages of the production chain, including pre-harvest, processing, storage in silos and piles, and post-harvest storage in bags [2,3]. Potentially mycotoxigenic fungi can colonize plants during crop development and, under favorable environmental conditions, produce mycotoxins before harvest [4]. These toxins may persist in silages [5] and occur in freshly harvested and stored grains [6]. Among the most relevant mycotoxins are aflatoxins, trichothecenes, ochratoxin A, fumonisins, and zearalenone [7]. In animals, exposure to mycotoxins may impair productive performance and ruminal function and increase susceptibility to infections [2,8,9].

Among these compounds, fumonisins are important contaminants of corn and corn-based feeds. Fumonisin B1 (FB1) and fumonisin B2 (FB2) are the predominant fumonisins detected in corn silage and are mainly produced by Fusarium verticillioides and Fusarium proliferatum [7]. Fumonisins can be present during crop development [4], in silage [5] and in stored grains [6], indicating that exposure through contaminated feed is a relevant concern for livestock production. Fumonisin exposure has been associated with inflammation, oxidative stress, and tissue injury, including hepatic alterations and impairment of antioxidant defenses [10,11,12]. Importantly, FB1 undergoes limited degradation in the rumen, which may allow part of the ingested toxin to reach the post-ruminal sections of the gastrointestinal tract and contribute to toxic effects in ruminants [13]. Therefore, strategies to reduce the adverse effects of dietary mycotoxins are of particular interest in ruminant production systems.

Several approaches have been investigated to mitigate the effects of mycotoxins in animal feed, including the use of antimycotoxin additives and adsorbent compounds [9,14,15,16]. Among mineral adsorbents, sodium aluminosilicates have been investigated because of their ability to interact with mycotoxins through physicochemical interactions between the mineral surface and toxin molecules [17,18,19,20]. These interactions may reduce the bioavailability of certain mycotoxins in the gastrointestinal tract, although the efficacy of mineral adsorbents can vary according to the type of mineral, toxin, and experimental conditions. In addition to mineral adsorbents, marine macroalgae have attracted interest as feed ingredients because of their nutritional composition and their content of bioactive compounds, including minerals, proteins, polysaccharides, pigments, fatty acids, and compounds with antioxidant, antimicrobial, and anti-inflammatory properties [21]. Recent evidence indicates that algae may also contribute directly to mycotoxin mitigation, as algae-based formulations have shown the ability to adsorb fumonisin B1 in vitro, potentially through interactions between mycotoxins and polysaccharides present in algal cell walls [22]. Thus, combining mineral adsorbents with marine macroalgae may represent a complementary approach to mitigating the biological effects of dietary mycotoxins. However, although previous in vitro studies have demonstrated the potential of algae-based formulations to adsorb fumonisin B1 [22], evidence regarding the in vivo efficacy of formulations combining calcium–sodium aluminosilicate and marine macroalgae remains limited, particularly in growing lambs. Furthermore, it remains unclear whether such combinations can attenuate the adverse effects of dietary fumonisin on productive performance, metabolic responses, oxidative status, and hematological parameters under prolonged exposure. Investigating this combination in growing lambs is therefore relevant to determine whether its proposed properties translate into measurable biological benefits under practical feeding conditions.

We hypothesized that dietary inclusion of an antimycotoxin additive combining calcium–sodium aluminosilicate and marine macroalgae would attenuate the adverse effects of fumonisin exposure in growing lambs. Therefore, this study evaluated the effects of this formulation on productive performance, serum biochemical variables, oxidative status, and hematological parameters in confined growing lambs fed fumonisin-contaminated diets.

## 2. Materials and Methods

### 2.1. Fumonisin Inoculum, Quantification and Antimycotoxin Additive

#### 2.1.1. Inoculum

The fumonisins used in this experiment were produced by Micotech, a laboratory located in Piracicaba, São Paulo, Brazil, using a fungal isolate of *Fusarium verticillioides*. Fermentation was carried out in 500 mL Erlenmeyer flasks, to which 100 g of cracked corn and 40 mL of distilled water were added. Flasks were autoclaved at 121 °C for 30 min (CS-75, Prismalab, Rio de Janeiro, RJ, Brazil) and then left at room temperature. The autoclaved material was inoculated with 2 mL of conidial suspension (1 × 10^8^ conidia per mL). The suspension was obtained from colonies of *Fusarium verticillioides* growing on potato dextrose agar for 10 days at 25 °C. After inoculation, the vials were kept static for 21 days at a controlled temperature (25 °C). Subsequently, the fermented material was dried in an oven at 57 °C and ground in a mill with a sieve < 0.85 mm to artificially contaminate the feed. The challenge dose stipulated for this study had a contamination level in the diet of approximately 30 ppm of fumonisins.

#### 2.1.2. Quantification

For fumonisin analyses, samples of feed were ground to <0.85 mm and one gram of the ground material was transferred to a 50 mL test tube. We added 10 mL of ultrapure water and 10 mL of acetonitrile/acetic acid (CH_3_CN:CH_3_COOH) [99.5:0.5, *v*/*v*], and the test tube was placed in a mechanic shaker for 30 min. A mixture of 4 g of MgSO_4_ and 1 g of NaCl was added and the tube was vigorously shaken by hand for 10 s. The solution was then centrifuged for 15 min at 10,000× *g*, at 4 °C, and 2.5 mL of supernatant was transferred to a capped glass test tube, to which 2.5 mL of hexane was added. The solution was shaken for 2 h and then centrifuged at 1000× *g*, at 20 °C, for 1 min. From the lower phase (acetonitrile), 1 mL was withdrawn and dried with a nitrogen (N) stream at 40 °C. The reconstitution was performed with 100 µL of methanol in an ultrasonic bath for 10 s and a test tube mixer for 10 s after adding 100 µL of ultrapure water. Ten microliters were injected in chromatographic system. Detection and quantification of mycotoxins were performed with high-performance liquid chromatography coupled with tandem mass spectrometry (Waters, Milford, MA, USA). Chromatographic separation was carried out using the Acqulty UPLC System (Waters, Waters, Milford, MA, USA) equipped with a 100 × 2.1 mm, 1.7 µm Acquity UPLC BEH C18 column, (Waters, Milford, MA, USA). The column was maintained at 40 °C and the injection volume was 10 µL. The mobile phase consisted of 0.1% formic acid in water (A) and 0.1% formic acid in acetonitrile (B). The acetonitrile (B) concentration was raised gradually from 10% to 90% within 12 min, brought back to the initial conditions at 0.1 min, and allowed to stabilize for 3 min. The mobile phase was delivered at a flow rate of 0.4 mL/min. The LC system was coupled with a Xevo TQS tandem mass spectrometer (Waters, Milford, MA, USA), equipped with a turbo-ion electrospray (ESI) ion source. The mass spectrometer was operated as scheduled multiple reaction monitoring (MRM) in positive mode. The data obtained by the mass spectrometer are shown in Table S1.

Mycotoxin quantification was carried out using matrix-matched calibration curves with 6 points of concentration (3.7 to 899.1 ng/mL), using uncontaminated extracts of the control diet. Recovery rates for each fumonisin were defined as the mean value obtained from five recovery tests using two distinct contamination levels. The tests were conducted using 1 g of the control diet (free from natural contamination), to which appropriate volumes of fumonisin B1 and B2 standards were added to achieve final contamination levels of 2 and 20 ppm for FB1, and 1 and 10 ppm for FB2. The observed recovery rates ranged from 84% to 91% and from 82% to 87%, respectively. Coefficients of variation were calculated based on the recovery tests and considered representative of the method’s variability. The method exhibited variability ranging from 9% to 14% for FB1 and from 12% to 16% for FB2. The limit of quantification was defined as the concentration of the lowest point on the calibration curve (3.7 ng/mL), resulting in a sample contamination level of 74 ppb. Samples with contamination levels exceeding the highest point on the calibration curve were appropriately diluted for quantification. The final contamination level observed in the contaminated diets is presented in Table S2.

#### 2.1.3. Additive

In this study, a technological additive (ROIMAX^®^, Akros, Chapecó, SC, Brazil), formulated from calcium–sodium aluminosilicate (87.5%) and marine macroalgae (12.5%), was tested.

### 2.2. Experimental Design

The experiment was conducted in the ovine facility of the Experimental Farm of the Centro de Educação Superior do Oeste (FECEO). Thirty-two Lacaune lambs, male and two months of age, with an average body weight of 18 kg, were used. At the beginning of the experiment, the animals were weighed and homogeneously allocated into four groups of eight animals each: control (without addition of mycotoxin or antimycotoxin additive), control + additive (3 kg/t of ROIMAX^®^ added to the diet), fumonisin (33.94 ppm of fumonisin added to the diet), and fumonisin + additive (33.94 ppm of fumonisin + 3 kg/t of ROIMAX added to the diet). The mean fumonisin concentration in the contaminated feed was 33.94 mg/kg, calculated from the analytical results presented in Supplementary Table S2.

Each animal was housed in an individual (a total floor area of 2.0 m^2^ per animal) pen throughout the experimental period to control food supply and leftovers. Both fumonisin and ROIMAX were added to the concentrate during its production at the FECEO experimental feed mill.

The diet was formulated according to the animals’ nutritional requirements. The total mixed ration (TMR) consisted of concentrate and corn silage supplied at 2.5% and 2.0% in DM of the lambs’ body weight, respectively. The diet contained 51.1% dry matter and 71.9% total digestible nutrients. Its chemical composition was 14.6% crude protein, 30.2% neutral detergent fiber, 35.9% starch, and 3.0% ether extract. To ensure the intake of fumonisin and the additive, the lambs received the concentrate first, followed by corn silage 30 min later, with the aim of stimulating the complete intake of the mycotoxin and ROIMAX. Feed was provided twice daily, at 08:00 and 16:00 h.

### 2.3. Data and Sample Collection

Sample collection and body weight measurements were performed on days 1, 14, 28, and 42 of the experimental period. Body weight was measured using a digital scale after a 12 h fasting period to monitor animal performance. Body weight gain and average daily gain (ADG) were calculated from these measurements. Feed intake was measured daily by weighing feed refusals in the morning before fresh feed was provided. Feed efficiency (ADG/DMI) and feed conversion ratio (DMI/ADG) were calculated based on these measurements. The individual lamb was considered the experimental unit for all evaluated variables, including dry matter intake (DMI), feed conversion ratio (FCR), and feed efficiency, as animals were housed individually and feed intake was measured separately for each animal.

Blood samples were collected from the jugular vein into tubes without anticoagulant. The blood samples were kept in a thermal box at temperatures between 2 and 8 degrees until complete blood count analysis. The tubes were centrifuged at 7000 rpm for 10 min to separate the serum for biochemical analyses. The supernatant was transferred to labeled 1.5 mL microtubes and stored at −20 °C until analysis.

### 2.4. Serum Biochemistry

Serum concentrations of total protein (TP), glucose, albumin, creatine kinase (CK), cholesterol, and urea, as well as activities of alanine aminotransferase (ALT), gamma-glutamyl transferase (GGT), aspartate aminotransferase (AST), were determined using an automated analyzer (EXC 200, Zybio Inc., Chongqing, China) and commercial kits (Analisa^®^, Analisa Diagnóstica Ltda., Belo Horizonte, MG, Brazil). Globulin concentrations were calculated as the difference between total protein and albumin concentrations.

### 2.5. Complete Blood Count

The complete blood count was performed using an automated hematology analyzer (Z4 VET, Equip^®^, Barueri, SP, Brazil). The following parameters were determined: erythrocyte count, hemoglobin concentration, hematocrit, platelet count, total leukocyte count, lymphocyte count, granulocyte and neutrophil counts, and the combined count of eosinophils, basophils, and monocytes.

### 2.6. TBARS and ROS Levels

Lipid peroxidation in serum was evaluated by quantifying thiobarbituric acid-reactive substances (TBARSs), according to the method described by Jentzsch et al. [23]. Briefly, 20 µL of serum was mixed with 55 µL of distilled water, 100 µL of orthophosphoric acid (0.2 M), and 25 µL of thiobarbituric acid (TBA, 0.1 M). The mixture was incubated for 45 min at 90 °C. Subsequently, absorbance was measured spectrophotometrically at 532 nm using a SpectraMax iD3 microplate reader (Molecular Devices, San Jose, CA, USA). The results were expressed as nmol of malondialdehyde (MDA)/mL.

Serum ROS-related fluorescence was assessed using the 2′,7′-dichlorofluorescein diacetate (DCFH-DA) assay, following the method described by Ali et al. [24]. Briefly, 10 µL of serum was incubated with 10 µL of DCFH-DA solution and 230 µL of distilled water. The mixture was incubated for 30 min at 37 °C in a black microplate. Fluorescence resulting from the formation of dichlorofluorescein (DCF) was measured at excitation and emission wavelengths of 485 and 525 nm, respectively, using a SpectraMax iD3 microplate reader (Molecular Devices, San Jose, CA, USA). The results were expressed as relative fluorescence units per milligram of protein (RFU/mg protein).

### 2.7. Data Analysis

Data normality was assessed using the Shapiro–Wilk test, while homogeneity of variances was evaluated using Levene’s test. Skewness and kurtosis were also assessed, and linearity was evaluated by linear regression. The four dietary combinations were considered individual treatments in the statistical model because the primary objective was to compare their biological responses, particularly the effects of additive inclusion in lambs receiving fumonisin-contaminated diets. Based on these preliminary assessments, the data were analyzed using the MIXED procedure of SAS (SAS Institute Inc., Cary, NC, USA; version 9.4). Average daily gain (ADG), feed conversion ratio, feed efficiency, and blood variables were analyzed considering treatment, day, and the treatment × day interaction as fixed effects in a completely randomized design. Body weight and dry matter intake were analyzed as repeated measures, with treatment, day, and the treatment × day interaction as fixed effects, and animal considered the subject of repeated measurements. Day 1 values for each variable were initially included as covariates in the respective models; however, the covariate was removed from the final models because its effect was not significant (*p* > 0.05). Means were compared using Tukey’s test. The results are presented as means ± standard error of the mean (SEM), and statistical significance was declared at *p* ≤ 0.05.

## 3. Results

Based on the lambs’ daily concentrate intake and the amount of fumonisin in the diet, the daily fumonisin intake was calculated to be approximately 16.5 mg. However, it is important to note that no apparent clinical signs were observed in the sheep during the experimental period.

### 3.1. Animal Performance

There was no effect of the different diets or the diet × day interaction on lamb body weight throughout the experimental period (*p* > 0.05; Table 1). Similarly, body weight gain from days 1 to 14 and dry matter intake (DMI) during the evaluated period did not differ among treatments (*p* > 0.05). However, from days 15 to 42, the fumonisin challenge significantly impaired animal performance. Lambs in the Fumo group had a 20.0% reduction in body weight gain (5.04 vs. 6.30 kg) and average daily gain (ADG; 0.180 vs. 0.225 kg/day) compared with the control group (*p* = 0.01). Likewise, feed efficiency was 22.6% lower, whereas feed conversion ratio was 26.8% higher in the Fumo group than in the control group (*p* = 0.01).

The inclusion of the additive in the diet without fumonisin (Cont + Add) resulted in numerically greater body weight gain compared with the control and Fumo + Add groups. In the fumonisin-contaminated diet, inclusion of the additive (Fumo + Add) partially attenuated the negative effects of the mycotoxin, resulting in values similar to those observed in the control group for body weight gain, ADG, feed efficiency, and feed conversion ratio (*p* > 0.05). However, body weight gain in the Fumo + Add group remained lower than that observed in the Cont + Add group.

### 3.2. Serum Biochemistry and Oxidative Stress

Serum concentrations of albumin, globulin, total protein, cholinesterase (ChE), alanine aminotransferase (ALT), and urea were not affected by diets (*p* > 0.05; Table 2). In contrast, fumonisin exposure significantly altered several biochemical parameters. Serum creatine kinase (CK) activity was 98.2% higher in the Fumo group than in the control group (652 vs. 329 U/L; *p* = 0.001). In addition, fumonisin exposure was associated with a 15.1% reduction in serum cholesterol concentration and increases of 9.7% and 7.7% in serum GGT and AST activities, respectively, compared with the control group (*p* = 0.05).

Markers of oxidative stress were also affected by treatment. Serum TBARS and ROS concentrations were higher in lambs from the Fumo group than in those from the control group (*p* ≤ 0.05). In the Fumo + Add group, TBARS and ROS concentrations were intermediate between those observed in the Fumo and control groups and did not differ statistically from the control group.

Similarly, the inclusion of the antimycotoxin additive in the fumonisin-contaminated (Fumo + Add) diet resulted in intermediate values for CK, cholesterol, GGT, and AST, with values statistically similar to those observed in the control group (Table 2). These results indicate that the additive attenuated some of the biochemical and oxidative alterations associated with fumonisin exposure.

**Figure 1:**
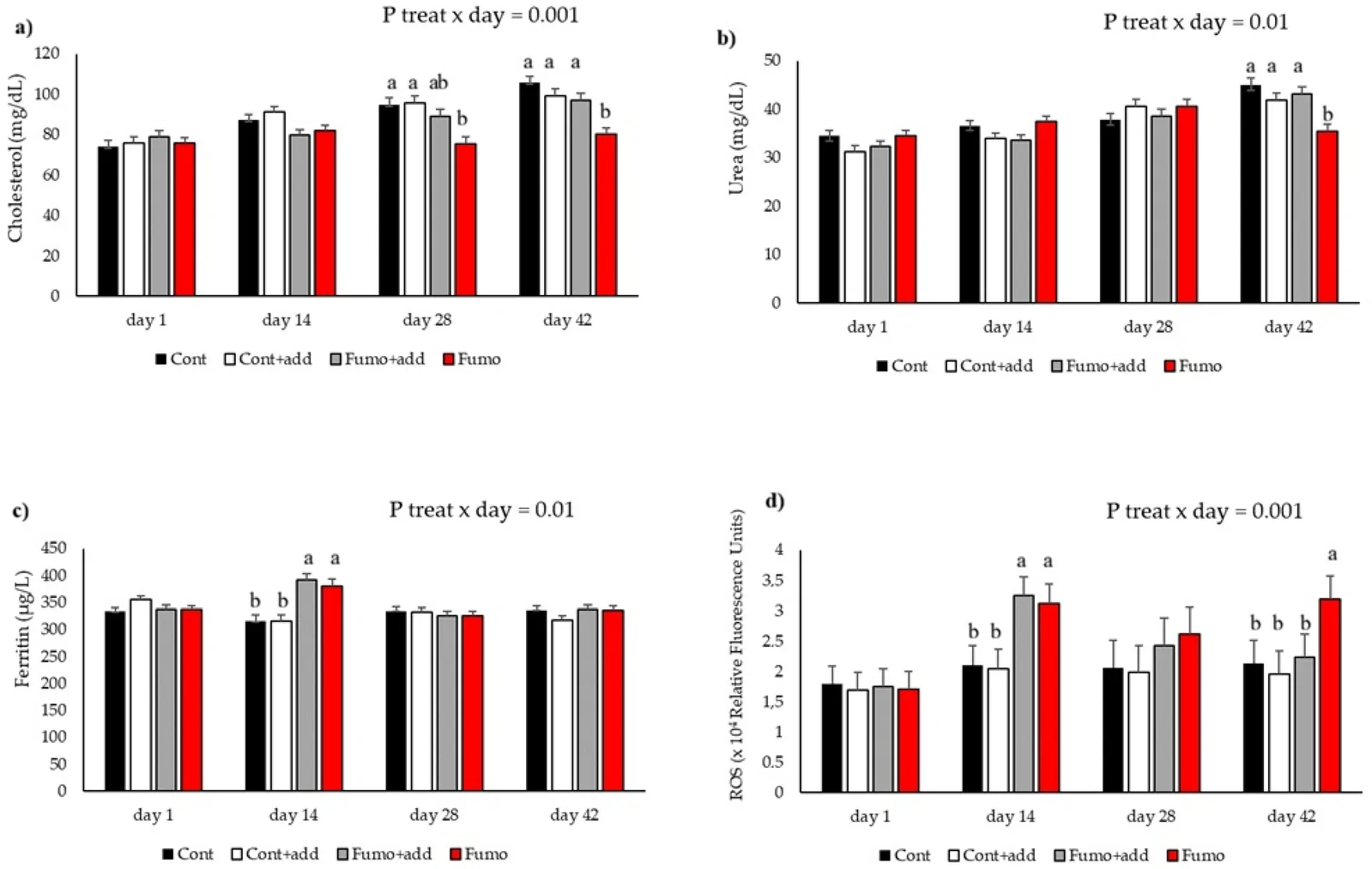
The effects of inclusion of an antimycotoxin additive on serum concentrations of (**a**) cholesterol, (**b**) urea, (**c**) ferritin, and (**d**) reactive oxygen species (ROS) in lambs challenged with fumonisin throughout the experimental period. Cont = control; Cont + Add = control + additive; Fumo + Add = fumonisin + additive; Fumo = fumonisin. Data are presented as the means ± SEM. Different letters indicate differences among treatments within the same day (*p* ≤ 0.05). P treatment × day indicates the effect of the treatment × day interaction.

A significant treatment × day interaction was observed for serum cholesterol (*p* = 0.001), urea (*p* = 0.01), ferritin (*p* = 0.01), and reactive oxygen species (ROS; *p* = 0.001) concentrations. Serum cholesterol concentrations were similar among groups up to day 14. However, on days 28 and 42, lambs in the Fumo group had lower serum cholesterol concentrations than those in the control group, whereas the Cont + Add and Fumo + Add groups maintained values similar to those of the control group (Figure 1a). Serum urea concentrations did not differ among treatments up to day 28. At the end of the experimental period (day 42), however, lambs in the Fumo group had lower serum urea concentrations than those in the control group, whereas inclusion of the additive, both in the absence and presence of fumonisin, maintained values similar to those observed in the control group (Figure 1b). For ferritin, differences among treatments were observed only on day 14, when the Fumo and Fumo + Add groups had higher concentrations than the control group. From day 28 onward, these differences were no longer observed, indicating a normalization of ferritin concentrations over the experimental period (Figure 1c).

Serum ROS concentrations also showed a significant treatment × day interaction. On day 14, lambs in the Fumo and Fumo + Add groups had higher ROS concentrations than those in the control and Cont + Add groups. However, on day 42, only the Fumo group maintained elevated ROS concentrations, whereas the Fumo + Add group showed values similar to those of the control group. This pattern suggests that inclusion of the additive attenuated the increase in oxidative stress-associated biomarkers observed in response to fumonisin exposure over the experimental period (Figure 1d).

**Figure 2:**
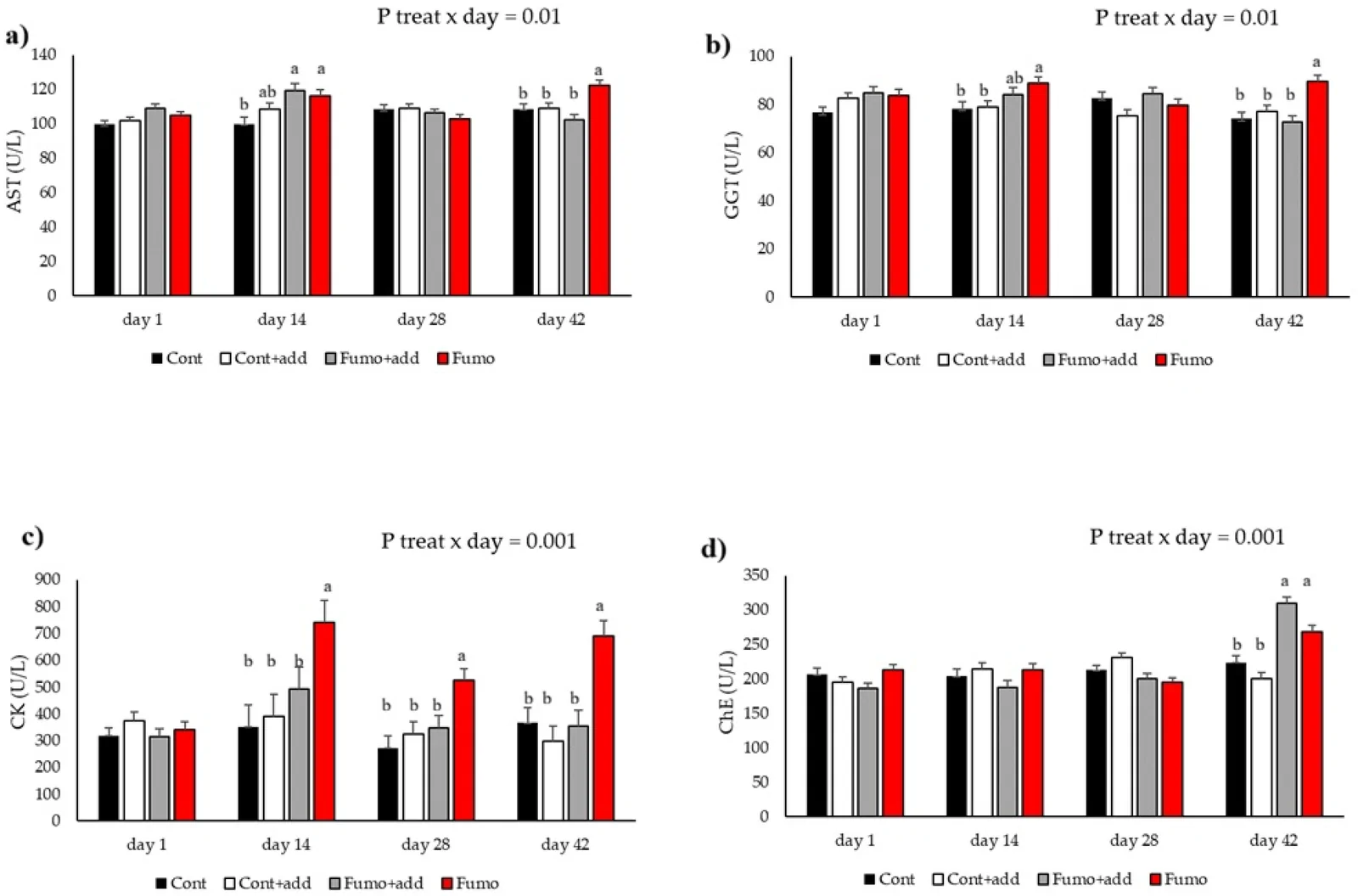
Serum activities of (**a**) aspartate aminotransferase (AST), (**b**) gamma-glutamyl transferase (GGT), (**c**) creatine kinase (CK), and (**d**) cholinesterase (ChE) in lambs subjected to different treatments throughout the experimental period. Cont = control; Cont + Add = control + additive; Fumo + Add = fumonisin + additive; Fumo = fumonisin. Data are presented as the means ± SEM. Different letters indicate differences among treatments within the same day (*p* ≤ 0.05). P treatment × day indicates the effect of the treatment × day interaction.

A significant treatment × day interaction was also observed for the serum activities of AST (*p* = 0.01), GGT (*p* = 0.01), CK (*p* = 0.001), and ChE (*p* = 0.001). AST activities were similar among groups at the beginning of the experiment; however, on day 14, lambs in the Fumo and Fumo + Add groups had higher activities than those in the control group. On day 42, only the Fumo group maintained higher serum AST activity than the control group, whereas the other groups showed values similar to those of the control group (Figure 2a). For GGT, differences among treatments were also observed on days 14 and 42. At both time points, lambs in the Fumo group had higher serum GGT activity than those in the control group, whereas animals receiving the additive in the fumonisin-contaminated diet (Fumo + Add) showed intermediate values that were similar to those of the control group (Figure 2b).

Serum CK activity showed the most pronounced changes among the enzymes evaluated. On days 14, 28, and 42, lambs in the Fumo group had higher CK activity than those in the control group, whereas the Fumo + Add group showed a less pronounced increase, suggesting an attenuating effect of the additive on the increase in CK activity associated with fumonisin exposure (Figure 2c). For cholinesterase (ChE), differences among treatments were observed only at the end of the experimental period. On day 42, the Fumo + Add group had the highest serum ChE activity, followed by the Fumo group, whereas the Control and Cont + Add groups had the lowest values (Figure 2d).

### 3.3. Hematological Parameters

Most hematological variables were not affected by treatment (*p* > 0.05; Table 3). Erythrocyte, hemoglobin, platelet, monocyte, and granulocyte counts did not differ among treatments, and no treatment × day interaction was observed for these variables (*p* > 0.05). Hematocrit was the only erythrocytic variable affected by treatment (*p* = 0.05), with the Fumo group showing a lower value than the control group (33.8 vs. 36.3%; Table 3). The Cont + Add and Fumo + Add groups presented intermediate values and did not differ from either the control or Fumo groups.

**Figure 3:**
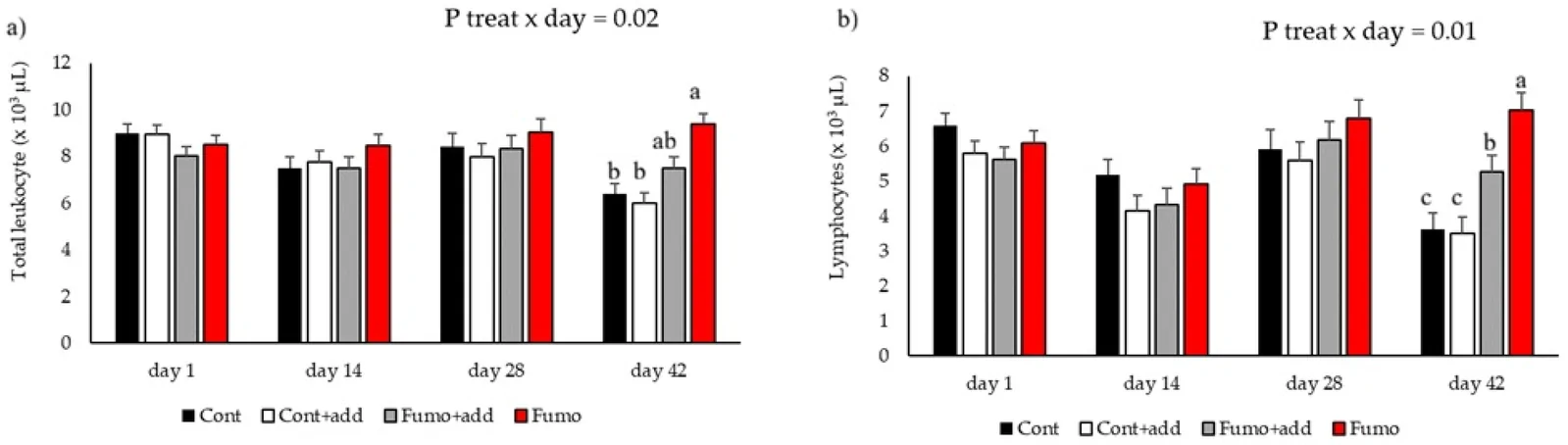
Total counts of (**a**) leukocytes and (**b**) lymphocytes in lambs subjected to different treatments throughout the experimental period. Cont = control; Cont + Add = control + additive; Fumo + Add = fumonisin + additive; Fumo = fumonisin. Data are presented as the means ± SEM. Different letters indicate differences among treatments within the same day (*p* ≤ 0.05).

A significant treatment × day interaction was observed for leukocyte and lymphocyte counts (*p* = 0.02 and *p* = 0.01, respectively; Table 3). Leukocyte counts did not differ among treatments during the first 28 days of the experiment. However, on day 42, lambs in the Fumo group had higher leukocyte counts than those in the control group, whereas the Cont + Add and Fumo + Add groups showed intermediate values that did not differ from the control group (Figure 3a). A similar pattern was observed for lymphocytes. No differences among treatments were detected up to day 28; however, on day 42, the Fumo group had a higher lymphocyte count than the control group. The Fumo + Add group showed intermediate values that were similar to those observed in the control group (Figure 3b).

## 4. Discussion

The results of the present study demonstrate that dietary exposure to fumonisin at 33.9 ppm adversely affected the performance and metabolic status of growing lambs, with the most evident effects occurring from days 15 to 42. During this period, lambs exposed to fumonisin without the antimycotoxin additive had lower body weight gain, average daily gain, and feed efficiency, together with a higher feed conversion ratio, despite no differences in dry matter intake. This pattern indicates that the negative effect of fumonisin was not primarily associated with feed intake but rather with the efficiency with which ingested nutrients were utilized. Such a response is consistent with the biological effects attributed to fumonisins, which can interfere with cellular metabolism, tissue integrity, and nutrient utilization after prolonged exposure [25,26]. Importantly, the absence of an effect on dry matter intake suggests that the reduction in growth was not simply a consequence of reduced appetite. Instead, the lower productive efficiency observed in the Fumo group may reflect metabolic costs associated with the toxic response and/or impairment of gastrointestinal and systemic functions. Previous studies have also reported reduced growth performance in animals exposed to fumonisins or diets contaminated with multiple mycotoxins, supporting the relevance of these compounds even when overt clinical signs are absent [16,27].

The delayed appearance of the performance response is also biologically relevant. No differences in body weight gain were detected during the first 14 days, whereas clear differences emerged between days 15 and 42. This temporal pattern may indicate that the effects of continuous fumonisin exposure became more apparent as exposure accumulated. Fumonisins are not considered to exert their toxicity through a single nonspecific mechanism; rather, their major biological effect involves disruption of sphingolipid metabolism. Fumonisin B1, the predominant fumonisin in contaminated feed, structurally resembles sphingoid bases and inhibits ceramide synthase, resulting in reduced synthesis of ceramides and complex sphingolipids and accumulation of sphinganine and sphingosine and their phosphorylated derivatives [25]. Because sphingolipids participate in cell membrane structure, intracellular signaling, proliferation, apoptosis, and inflammatory responses, prolonged disruption of this pathway may affect multiple tissues and physiological processes. Therefore, the progressive reduction in productive efficiency observed in the present study may represent the cumulative consequence of cellular and metabolic disturbances rather than an immediate reduction in feed intake.

Alterations in gastrointestinal integrity may also contribute to the lower efficiency of nutrient utilization associated with fumonisin exposure. Experimental studies in pigs have shown that fumonisins can impair intestinal morphology and epithelial integrity, including reduced villus height, villus fusion and atrophy, flattening of enterocytes, and apical epithelial damage [26,28]. Such alterations may compromise the absorptive capacity of the intestinal epithelium and consequently affect nutrient absorption. However, intestinal morphology, permeability, or specific biomarkers of enterocyte injury were not evaluated in the present experiment. Therefore, an intestinal mechanism cannot be directly demonstrated here and should be considered a possible explanation rather than a confirmed mechanism. Future studies combining performance measurements with intestinal histology, permeability markers, and biomarkers such as intestinal fatty acid-binding protein could help determine whether impaired intestinal integrity contributes to the reduction in feed efficiency observed during prolonged fumonisin exposure.

The biochemical results provide additional evidence that fumonisin exposure affected systemic metabolism. The most pronounced alteration was the increase in serum creatine kinase (CK) activity in the Fumo group, which was almost twice that observed in the control group. Increased CK activity is compatible with altered cellular integrity and leakage of intracellular enzymes into the circulation. Because CK is particularly abundant in skeletal muscle, the increase may be consistent with an alteration in muscle cellular integrity; however, CK alone does not establish the presence, severity, or specific origin of tissue injury. Thus, the present results should not be interpreted as definitive evidence of fumonisin-induced muscle damage. A similar increase in CK was reported in lambs exposed to a diet contaminated with multiple mycotoxins, including fumonisin, particularly after prolonged exposure [16]. Since this increase was also found in this study, the recurrence of this response across lamb studies suggests that CK may be a useful indicator of systemic cellular stress during prolonged mycotoxin exposure, although complementary markers and histopathological assessments would be necessary to establish tissue specificity.

The increased activities of AST and GGT in fumonisin-exposed lambs further indicate that prolonged exposure affected biochemical parameters associated with tissue and hepatic or hepatobiliary function. Fumonisins are known to affect the liver, and disruption of sphingolipid metabolism is considered an important component of fumonisin-associated hepatotoxicity [25]. However, the interpretation of the present enzyme profile requires caution. AST is not liver-specific and can also originate from skeletal muscle and other tissues, whereas ALT was not affected by treatment in the present study. Therefore, the simultaneous increase in AST, GGT, and CK is better interpreted as evidence of a broader systemic response compatible with hepatic/hepatobiliary and cellular alterations rather than as definitive proof of isolated liver injury. This interpretation is consistent with previous observations in lambs exposed to multiple mycotoxins, in which increases in AST and GGT accompanied other metabolic and oxidative alterations [16].

The lower concentration of cholesterol in serum observed in fumonisin-exposed lambs may also be related to the metabolic consequences of fumonisin toxicity. The liver plays a central role in cholesterol synthesis, transport, and metabolism, and fumonisin exposure has been shown to disrupt hepatic lipid homeostasis and cholesterol-related pathways [16,29]. More importantly, because ceramide synthase inhibition disrupts sphingolipid metabolism and membrane lipid homeostasis, changes in circulating cholesterol may reflect broader alterations in lipid metabolism rather than a specific alteration in cholesterol synthesis alone [25]. The fact that cholesterol differences became evident mainly after day 28 supports the possibility of a time-dependent metabolic response to continued exposure. Nevertheless, cholesterol is a nonspecific metabolic variable, and the present study did not include direct measurements of hepatic lipid metabolism or sphingolipid metabolites. Therefore, this interpretation should remain mechanistic rather than definitive.

A similar time-dependent pattern was observed for serum urea. Although urea concentrations were not different among treatments during most of the experimental period, the Fumo group had lower values than the control group on day 42. Changes in serum urea have previously been reported in animals exposed to fumonisins, including lambs, although the direction and magnitude of the response may vary according to dose, duration of exposure, and experimental conditions [30]. In ruminants, circulating urea reflects the integrated metabolism of nitrogen, including hepatic conversion of absorbed ammonia and amino acid-derived nitrogen to urea, as well as subsequent urea recycling and excretion [31]. Therefore, the lower serum urea observed in the present study may be associated with alterations in nitrogen metabolism, protein utilization, or hepatic ureagenesis. However, this interpretation remains speculative because nitrogen balance, ruminal ammonia, amino acid metabolism, and direct measures of hepatic urea synthesis were not evaluated. Importantly, the reduction in serum urea should not be interpreted as evidence of renal dysfunction, particularly because other indicators of renal function were not altered or were not evaluated in the present study. The absence of treatment effects on total protein, albumin, and globulin also suggests that the lower urea concentration was not accompanied by a generalized alteration in circulating protein status.

The increase in serum ferritin observed on day 14 in the fumonisin-exposed groups, followed by the absence of treatment differences at later time points, indicates a transient treatment-related response during the experimental period. Ferritin is primarily involved in iron storage and homeostasis, but its circulating concentration can also increase in response to inflammation and oxidative stress, reflecting its role as an acute-phase and stress-responsive protein [32,33,34]. Ferritin expression may be induced by inflammatory mediators and reactive oxygen species, contributing to the sequestration of iron and limiting its availability for redox reactions [32,34]. Thus, the transient increase observed on day 14 could be compatible with an early systemic response to the dietary fumonisin challenge. However, this interpretation should be considered cautiously because inflammatory cytokines, serum iron, transferrin, and other acute-phase proteins were not measured in the present study. Consequently, the increase in ferritin cannot be specifically attributed to inflammation, altered iron metabolism, or oxidative stress. The disappearance of treatment differences at subsequent time points may indicate that this response was not sustained throughout the 42-day exposure period.

Oxidative imbalance appears to be an important component of fumonisin toxicity. The higher TBARS and ROS-related fluorescence observed in fumonisin-exposed lambs is consistent with previous evidence that fumonisins can promote oxidative stress, possibly as a result of disrupted sphingolipid metabolism and cellular homeostasis [25,35]. Similar increases in ROS and TBARSs were reported in lambs exposed to diets contaminated with multiple mycotoxins, whereas an antimycotoxin additive attenuated these responses [16]. In the present study, the lower ROS-related fluorescence in lambs receiving the additive suggests attenuation of the oxidative response associated with fumonisin exposure. However, because antioxidant enzymes and other components of the antioxidant system were not evaluated, it cannot be determined whether this response resulted from reduced oxidant generation, preservation of antioxidant defenses, or both. Overall, the concurrent changes in TBARS and ROS-related fluorescence support an association between fumonisin exposure and altered redox status in growing lambs.

The hematological profile suggests a time-dependent systemic response to fumonisin exposure. The isolated reduction in hematocrit, in the absence of corresponding changes in erythrocyte count or hemoglobin, does not provide sufficient evidence of anemia. More importantly, the treatment × day interactions for leukocytes and lymphocytes, with higher counts in fumonisin-exposed lambs at the end of the experiment, indicate that the hematological response developed with prolonged exposure. Similar hematological responses have been reported in lambs exposed to mycotoxins, although the direction and magnitude of leukocyte changes vary with the type, dose, and duration of exposure [16,30]. The lower leukocyte and lymphocyte responses in the Fumo + Add group at the final evaluation may indicate attenuation of this systemic response by the additive. However, without measurements of cytokines or other inflammatory mediators, these hematological changes should be interpreted as evidence of an altered systemic immune response rather than as definitive evidence of inflammation.

A relevant finding of the present study was that inclusion of the antimycotoxin additive attenuated several alterations associated with dietary fumonisin exposure, particularly those involving productive performance, serum biochemical variables, and oxidative status. The additive consisted of calcium–sodium aluminosilicate and marine macroalgae, two components with potentially complementary properties. Mineral adsorbents may interact with certain mycotoxins in the gastrointestinal tract, although their binding efficacy depends on the physicochemical characteristics of both the adsorbent and the toxin [15,19]. Marine macroalgae, in turn, contain bioactive compounds with reported antioxidant and immunomodulatory properties [36]. Furthermore, previous in vitro research demonstrated fumonisin B1 adsorption by algae-modified clay formulations [22]. These findings provide a scientific rationale for investigating combinations of mineral and algal components; however, they do not establish that the same mechanisms occurred in the present experiment. Importantly, the additive was evaluated as a complete formulation, without separate assessment of its mineral and macroalgae fractions or direct measurement of fumonisin bioavailability. Consequently, the relative contribution of each component and the possible involvement of gastrointestinal adsorption or biological modulation cannot be determined from the present results. The observed attenuation of biochemical, oxidative, and performance-related alterations should therefore be attributed to the inclusion of the formulated additive rather than to a specific mechanism of action. Additionally, although the Control + Add group showed numerically greater performance for some variables, these findings do not establish an intrinsic growth-promoting effect. Overall, the results support the potential of the additive to partially mitigate the adverse responses associated with fumonisin exposure under the experimental conditions evaluated.

The attenuation of performance, biochemical, oxidative, and hematological alterations in the Fumo + Add group may be related to the combined properties of the additive components. The aluminosilicate fraction could have reduced the gastrointestinal availability of fumonisin through adsorption, thereby limiting toxin exposure. However, adsorption of fumonisins by mineral binders is variable and depends on both the characteristics of the adsorbent and the physicochemical properties of the toxin [15,19]. Thus, the improved responses observed in the present study are compatible with reduced fumonisin availability, but this mechanism cannot be confirmed because toxin recovery or bioavailability was not measured.

The marine macroalgae component may have provided an additional biological contribution. Marine macroalgae contain bioactive compounds with antioxidant and immunomodulatory properties that may help modulate responses to dietary challenges [36]. Importantly, recent in vitro evidence demonstrated that formulations combining clay and algae can adsorb fumonisin B1, with polysaccharides associated with algal cell walls proposed as potential binding sites for mycotoxins [22]. This finding is particularly relevant to the present study because the additive combines an aluminosilicate mineral fraction with marine macroalgae, providing a plausible basis for complementary adsorption and biological effects. The attenuation of oxidative and systemic responses observed in the Fumo + Add group is consistent with this interpretation. Nevertheless, the formulation evaluated by da Silva et al. [22] was not identical to that used in the present experiment, and their study was conducted in vitro. Furthermore, the mineral and macroalgae components were not evaluated separately in the present study, and fumonisin bioavailability was not measured. Therefore, the individual contribution of each component and the extent to which adsorption contributed to the observed biological responses cannot be established.

The attenuation of the oxidative response in the Fumo + Add group may also be related to the bioactive properties of the marine macroalgae component. Marine macroalgae contain compounds with antioxidant and immunomodulatory activities that may influence redox homeostasis [36,37]. Thus, the lower ROS-related fluorescence and TBARS response observed with additive inclusion could reflect not only reduced fumonisin availability through adsorption, but also direct or indirect modulation of oxidative status. However, because the mineral and macroalgae components were not tested separately, their individual contributions cannot be distinguished. The present results therefore support a potential complementary action of adsorption and biological modulation, rather than a specific mechanism. The numerically greater performance observed in the Control + Add group for some variables should be interpreted cautiously. Although this pattern may suggest that the additive had effects beyond mycotoxin mitigation, dry matter intake was not altered and the experiment was not designed to investigate mechanisms of performance enhancement under non-contaminated conditions. Therefore, this numerical response should not be interpreted as evidence of an intrinsic growth-promoting effect. In contrast, the more consistent pattern observed under fumonisin challenge, with attenuation of several performance and health-related alterations, provides stronger evidence for a protective effect of additive inclusion under the conditions tested.

Taken together, the present findings indicate that exposure to 33.9 ppm fumonisin affected growing lambs through alterations in productive efficiency, serum biochemical variables, redox status, and hematological responses. This overall pattern is consistent with the multifaceted toxicity of fumonisins, which is closely related to disruption of sphingolipid metabolism and consequent disturbances in cellular homeostasis and signaling [25,26,28,29,30,31,32,33,34,35,37]. The temporal pattern observed in several variables further suggests that some effects became more evident with continued exposure, supporting the relevance of exposure duration when evaluating fumonisin toxicity. The attenuation of several responses in the Fumo + Add group indicates that inclusion of the formulated additive partially mitigated the adverse effects associated with fumonisin exposure. This finding is compatible with the proposed action of mycotoxin adsorbents, which may reduce gastrointestinal exposure to toxins through binding, although adsorption efficacy varies according to the adsorbent and mycotoxin involved [15,19]. The potential contribution of marine macroalgae is also supported by evidence of antioxidant and immunomodulatory activities of algal compounds [36] and by recent in vitro evidence of fumonisin B1 adsorption by algae-modified clay formulations [22]. Nevertheless, because fumonisin bioavailability was not measured and the additive components were not evaluated separately, neither the mechanisms responsible for the observed responses nor the individual contributions of aluminosilicate and marine macroalgae can be established from the present findings.

## 5. Conclusions

Dietary exposure to fumonisin at 33.9 ppm impaired the productive performance of growing lambs, reducing body weight gain, average daily gain, and feed efficiency while increasing feed conversion ratio, without affecting dry matter intake. Fumonisin exposure was also associated with alterations in serum biochemical parameters, oxidative status, and hematological responses. The inclusion of the antimycotoxin additive based on aluminosilicate and marine macroalgae partially attenuated the negative effects on productive performance, while several biochemical and oxidative variables showed responses closer to those observed in control animals. These findings suggest that the formulated additive may help mitigate some of the adverse consequences associated with dietary fumonisin exposure in growing lambs under the experimental conditions evaluated, although complete protection was not demonstrated.

## Figures and Tables

**Table 1:** Growth performance of lambs challenged with fumonisin and receiving an antimycotoxin additive.

Variables	Cont	Cont + Add	Fumo + Add	Fumo	SEM	*p*-Treat ^1^	*p*-Treat × Day ^2^
Body weight, kg						0.32	0.46
d1	17.8	18.0	18.1	18.1	0.47		
d14	20.5	21.5	20.9	21.7	0.53		
d28	23.1	25.0	23.7	24.1	0.61		
d42	26.8	28.3	26.9	26.8	0.63		
Weight gain, kg							
d1–14	2.72	3.57	2.77	3.62	0.21	0.16	NE
d15–42	6.30 ab	6.78 a	6.03 b	5.04 c	0.22	0.01	NE
Average daily gain, kg							
d15–42	0.225 ab	0.242 a	0.215 b	0.180 c	0.06	0.01	NE
Dry matter intake, kg							
d1–14	0.56	0.59	0.57	0.58	0.02	0.92	0.95
d15–42	0.75	0.74	0.73	0.75	0.02	0.94	0.97
Feed efficiency, d15–42	0.314 ab	0.332 a	0.296 b	0.243 c	0.01	0.01	NE
Feed conversion, d15–42	3.40 bc	3.09 c	3.55 b	4.310 a	0.18	0.01	NE

Note ^1^. Treatment effect was considered significant when *p* < 0.05 and is indicated by different lowercase letters within the same row. Note ^2^. No treatment × day interaction was observed (*p* > 0.05). NE = not evaluated. Cont = control; Cont + Add = control + additive; Fumo + Add = fumonisin + additive; Fumo = fumonisin.

**Table 2:** Serum biochemical and metabolic parameters of lambs challenged with fumonisin and receiving an antimycotoxin additive.

Group	Albumin (g/dL)	CK (U/L)	Cholesterol (mg/dL)	ChE (U/L)	Globulin (g/dL)	Total Protein (g/dL)
Cont	3.43	329 b	95.9 a	227.4	2.08	5.52
Cont + Add	3.43	404 b	95.3 a	225.3	2.18	5.62
Fumo + Add	3.37	398 b	88.6 ab	233.0	2.13	5.50
Fumo	3.43	652 a	81.4 b	225.7	1.96	5.39
SEM	0.03	51.90	3.19	10.08	0.05	0.05
*p*-Value ^1^	0.892	0.001	0.050	0.910	0.580	0.530
	**GGT** **(U/L)**	**AST** **(U/L)**	**ALT** **(U/L)**	**Urea** **(mg/dL)**	**TBARS (nmol/mL)**	**ROS** **(RFU)**
Cont	78.4 b	105.7 b	23.4	39.8	2.45 b	2.09 b
Cont + Add	77.2 b	109.0 ab	24.4	38.8	2.39 b	1.99 b
Fumo + Add	80.5 ab	109.3 ab	23.8	36.4	2.56 ab	2.63 ab
Fumo	86.0 a	113.8 a	24.4	37.9	2.67 a	2.97 a
SEM	2.51	2.11	0.66	1.32	0.12	0.38
*p*-Value ^1^	0.050	0.050	0.790	0.135	0.05	0.03

Note ^1^: When the *p*-value is ≤0.05, the groups differ statistically for the variable, with the difference indicated by different letters in the same column. Cont = control; Cont + Add = control + additive; Fumo + Add = fumonisin + additive; Fumo = fumonisin.

**Table 3:** Hematological parameters of lambs challenged with fumonisin and receiving an antimycotoxin additive.

Variables	Cont	Cont + Add	Fumo + Add	Fumo	SEM	P-Treat	P-Treat × Day
Leukocytes (×10^3^ µL)	7.43	7.25	7.78	8.95	0.50	0.76	0.02
Lymphocytes (×10^3^ µL)	5.24	4.73	5.59	6.58	0.48	0.23	0.01
Monocytes (×10^3^ µL)	0.25	0.31	0.17	0.18	0.07	0.84	0.79
Granulocytes (×10^3^ µL)	2.35	2.15	2.08	2.20	0.17	0.92	0.88
Erythrocytes (×10^6^ µL)	11.9	11.9	11.6	10.9	0.26	0.45	0.47
Hematocrit (%)	36.3 a	35.6 ab	35.2 ab	33.8 b	0.69	0.05	0.07
Hemoglobin (mg/dL)	10.8	10.8	10.7	10.0	0.21	0.53	0.31
Platelets (×10^3^ µL)	404	397	394	395	21.33	0.74	0.61

Note 1. Treatment effect was considered significant when *p* < 0.05 and is indicated by different lowercase letters within the same row. Note 2. A significant treatment × day interaction was observed when *p* < 0.05; the corresponding results are presented in Figure 3. Cont = control; Cont + Add = control + additive; Fumo + Add = fumonisin + additive; Fumo = fumonisin.

## Data Availability

The data are held by the authors and can be made available upon request.

## Supplementary Materials

The following supporting information can be downloaded at https://www.mdpi.com/article/10.3390/ani16213342/s1: Table S1: Parameter data obtained by mass spectrometer; Table S2: Fumonisin levels in the concentrate fed to the lambs in the experiment.
