# Supplementary material for: The Effects of an Antimycotoxin Additive Based on Aluminosilicate and Marine Macroalgae in a Fumonisin-Contaminated Diet on the Performance and Health of Growing Lambs
Source: Animals (Basel). 2026 Oct 23;16(21):3342. doi: 10.3390/ani16213342 (PMC13762086; doi:10.3390/ani16213342)
Supplement: Supplementary file 1 [file animals-16-03342-s001.zip › animals-4614567-supplementary.pdf]

Tabel S1. Acquisition parameters data from mass spectrometer.

Variables	MRM Transiction	Dwell Time (s)	Cone Voltage (V)	Collision Energy (eV)
Aflatoxin B1	313.08>241.23	0.005	30	37
	313.08>285.39			23
IS Aflatoxin B1	330.00>300.90	0.005	30	23
Aflatoxin B2	315.10>259.05	0.005	30	28
	315.10>287.16			25
IS Aflatoxin B2	332.00>303.10	0.005	30	25
Aflatoxin G1	329.09>243.10	0.005	25	26
	329.09>283.00			26
IS Aflatoxin G1	346.00>257.00	0.005	25	26
Aflatoxin G2	331.05>245.05	0.005	25	30
	331.05>257.04			25
IS Aflatoxin G2	348.00>330.00	0.005	25	25
Ocratoxin A	404.20>221.10	0.005	25	30
	404.20>339.10			30
IS Ocratoxin A	424.00>250.00	0.005	25	25
Deoxynivalenol	297.19>231.18	0,037	20	10
	297.19>249.18			10
IS Deoxynivalenol	321.10>263.00	0.039	15	10
Zearalenone	319.20>185.20	0.005	20	19
	319.20>187.20			23
IS Zearalenone	337.00>199.10	0.005	20	19
Fumonisin B1	722.78>334.29	0.005	30	40
	722.78>352.22			35
IS Fumonisin B1	756.10>374.20	0.005	30	40
Fumonisin B2	706.57>318.22	0.005	30	40
	706.57>336.04			40
IS Fumonisin B2	740.20>358.20	0.005	30	36
Toxin T2	484.40>185.10	0.005	10	22
	484.40>215.10			22
IS Toxina T2	508.00>198.10	0.005	25	22

Table S2. Fumonisin levels in the concentrate fed to the lambs in the experiment.

Groups	Fumonisin B1 (ppb)	Fumonisin B2 (ppb)	Total concentration fumonisin (ppb) in the concentrate
Control	ND	ND	ND
Control + additive	ND	ND	ND
Fumonisin	26750	8465	35215
Fumonisin + additive	25126	7551	32677

ND: no-detected. Note: No presence of fumonisin was detected in the corn silage.
